# Impairment in Activities of Daily Living and Unmet Need for Care Among Older Adults: A Population-Based Study From Burkina Faso

**DOI:** 10.1093/geronb/gbab041

**Published:** 2021-03-14

**Authors:** Ben Brinkmann, Justine I Davies, Miles D Witham, Guy Harling, Till Bärnighausen, Mamadou Bountogo, Mark J Siedner, Lucienne Ouermi, Jana Junghanns, Boubacar Coulibaly, Ali Sié, Collin F Payne, Iliana V Kohler

**Affiliations:** 1 Heidelberg Institute of Global Health, Heidelberg University, Germany; 2 Institute of Applied Health Research, University of Birmingham, UK; 3 MRC/Wits Rural Public Health and Health Transitions Research Unit (Agincourt), Faculty of Health Sciences, University of the Witwatersrand, Johannesburg, South Africa; 4 AGE Research Group, NIHR Newcastle Biomedical Research Centre, Translational and Clinical Research Institute, Newcastle University and Newcastle upon Tyne Hospitals NHS Trust, UK; 5 Harvard Center for Population and Development Studies, Cambridge, Massachusetts, USA; 6 Institute for Global Health, University College London, UK; 7 Centre de Recherche en Santé de Nouna, Burkina Faso; 8 Africa Health Research Institute, KwaZulu-Natal, South Africa; 9 Department of Medicine, Massachusetts General Hospital, Harvard Medical School, Boston, USA; 10 School of Demography, Australian National University, Canberra, Australian Capital Territory, Australia; 11 Population Studies Center and Department of Sociology, University of Pennsylvania, Philadelphia, USA

**Keywords:** Activity impairment, Care need, Epidemiology, Health care, Sub-Saharan Africa

## Abstract

**Objectives:**

The importance of impairment in performing activities of daily living (ADL) is likely to increase in sub-Saharan Africa because few care options for affected people exist. This study investigated the prevalence of ADL impairment, the extent to which care need was met, and described characteristics of people with ADL impairment and unmet need in Burkina Faso.

**Methods:**

This study used data from the Centre de Recherche en Santé de Nouna Heidelberg Aging Study, a population-based study among 3,026 adults aged older than 40 years conducted in rural Burkina Faso. Information on 6 basic ADL items was sought, with a follow-up question asking whether care need was not met, partially met, or met. Bivariable correlations and multivariable logistic regression were used to determine sociodemographic and health characteristics associated with ADL impairment and unmet need.

**Results:**

ADL impairment of any kind was reported by 1,202 (39.7%) respondents and was associated with older age (adjusted odds ratio: 1.05 [95% CI: 1.04–1.06]), being a woman (1.33 [1.06–1.60]), and reporting depressive symptoms (1.90 [1.65–2.18]). Among those with ADL impairment, 67.8% had at least one unmet need. Severe ADL impairment was found in 202 (6.7%) respondents, who reported a lower prevalence of unmet need (43.1%). Severe ADL impairment was associated with depressive symptoms (2.55 [2.11–3.07]) to a stronger degree than any ADL impairment.

**Discussion:**

Prevalence of ADL impairment and unmet need was high in this setting. Variation in impairment across the population highlighted key groups for future interventions. Unmet need for care was highest in middle-aged adults, indicating a gap in care provision.

As is the case worldwide, populations in sub-Saharan Africa (SSA) and Burkina Faso are rapidly aging. The number of people in Burkina Faso aged older than 50 is estimated to more than double in the next 50 years (United Nations, Department of Economic and Social Affairs, & Population Division, 2019). Understanding the care need of older people in Burkina Faso is challenging, as there is very little evidence about physical function and health in later life (Berthe et al., 2012; Berthé et al., 2014) and no information regarding unmet need for care among functionally impaired people. The resources in this region are limited for older people and caregivers alike and the focus of care and research has traditionally been on earlier stages of the life course and on infectious diseases (Nachega et al., 2012).

The amount of research about functional health of older people from other SSA countries such as South Africa (Payne, Gómez-Olivé et al., 2017; Payne, Wade et al., 2017), Tanzania (Lewis et al., 2018), Nigeria (Ojagbemi et al., 2017), and Uganda (Yaya et al., 2020) is slowly increasing, but the majority of evidence still comes from high-income countries (HICs), where population aging is an established field of research. The very different socioeconomic, health, and cultural milieu present in Burkina Faso means that it cannot be assumed that similar patterns of either self-reported functional health impairment or care need will be present as is found in HICs or other SSA countries. The Centre de Recherche en Santé de Nouna (CRSN) Heidelberg Aging Study (CHAS) was specifically designed to extend the current research regarding functional health impairment and unmet need for care in Burkina Faso and SSA.

Physical, psychological, and cognitive impairments associated with aging have adverse effects on an older individual’s ability to perform everyday activities independently (Berkman et al., 1986; Fried & Guralnik, 1997). Activity impairment ultimately leads to a need for personal care from others. Long-term care (LTC) in SSA is traditionally performed by family members (Aboderin, 2010), and institutional care is rare (Gureje et al., 2006). The lack of institutional care and factors such as labor migration can lead to a gap in care provision (Harling et al., 2020), with the potential for rising numbers of older people to put an additional burden on informal care providers. Limited state provision of formal care leaves people with activity impairment who lack support from relatives a particularly vulnerable group (Atchessi et al., 2014).

The link between activity impairment and the use of formal and informal LTC services is best approached by investigating perceived or evaluated need for care (Andersen et al., 2013). An unmet need for care in people with activity impairment, be it perceived or evaluated, represents the gap between care need due to activity impairment and care provision. There is no universally agreed definition of unmet need for care in people living with a disability, but an approach based on activities of daily living (ADL) has been used in a range of studies from the National Long-Term Care Survey and studies using data from the English Longitudinal Study of Aging (Vlachantoni et al., 2011). In these studies, unmet care need in ADL-impaired persons was associated with higher mortality (He et al., 2015), higher frequencies of hospital admission (Xu et al., 2012) and readmission (DePalma et al., 2013), skin breakdown, weight loss, dehydration, and falls (LaPlante et al., 2004). Also, unmet need for care has been shown to be an important determinant of quality of life, mental health, and physical well-being in later stages of life (Allain et al., 1997; Gaugler et al., 2005).

However, external evaluation of unmet need for care may miss some perceived needs for care and those who receive help but have needs that are not adequately covered may be ignored (Lima & Allen, 2001). This additional need for care can be driven by a range of determinants, such as the caregivers’ inability to meet the needs, the desire of the care recipient to alleviate the burden put on the caregiver, or the wish to supplement the received informal care by formal care provision (Choi & McDougall, 2009). By asking people with ADL impairment directly about their perceived need for care, additional needs may be included. On the other hand, perception of unmet need for care is a subjective measure that may be influenced by cultural differences in expressing need (Branch, 2000) and may not necessarily mean that care need is not being met. Nevertheless, for this work, we base our theoretical framing of unmet need on the premise that if a respondent stated that they had unmet needs, they had them—the perception of unmet need is arguably more important than any external opinion (Dunatchik et al., 2019).

This is one of the first studies in SSA to explore the gradient of unmet, partially met, and met need for care among ADL-impaired older adults and how this gradient relates to sociodemographic factors, chronic morbidities, mental health, and cognitive impairment. Our measures of need are novel for the setting—in the past, unmet need for care in SSA has been determined indirectly (Harling et al., 2020) or dichotomized (Gureje et al., 2006). Including a range of unmet, partially met, and met need for care continues the approach of authors like Lima and Allen (2001), as it is important to not overlook those with objectively met need, but also to consider the additional perceived need for care.

This analysis aims to investigate the prevalence and correlates of (a) impaired ability to perform ADL at different degrees of severity and (b) unmet, partially met, and met need for care in a rural, poor, older adult population in SSA. Age, being female, low socioeconomic and educational status, cardiovascular disease, cardiovascular disease risk factors, depressive symptoms, and cognitive impairment have all been shown to be associated with higher levels of activity impairment (Chen et al., 2014; Harling et al., 2020; Santosa et al., 2016; Stuck et al., 1999; Tiago da Silva et al., 2012), and we hypothesize that we will find similarly positive associations with ADL impairment as well as unmet need for care in this low-income rural SSA population.

## Method

### Sample

The study used data from the CHAS, collected between May and July 2018. CHAS is a population-based study of adults aged 40 years and older based in a rural region in north-western Burkina Faso. It was conducted in the Nouna Health and Demographic Surveillance System (Sie et al., 2010), which consists of 58 villages centered around the town of Nouna. Of the 107,000 individuals living in the area in 2015, approximately 18,000 were older than the age of 40, out of which 3,996 individuals were selected in a stratified two-stage cluster random sampling approach. The sampling process is described in detail elsewhere (Witham et al., 2019).

Between May and July 2018, all sampled individuals were invited to participate in the study at their homes. Consenting participants completed a questionnaire on socioeconomic and demographic characteristics and their physical, cognitive, and mental health. Field workers were trained to administer the questionnaire, using French as the main language. Translations from French to the local language, Djula, were done within the training module and clearly communicated to all field workers verbally. Written Djula literacy is very limited in this setting, necessitating verbal rather than a written translation. Physical measurements such as grip strength and walk speed were measured by study staff (Witham et al., 2019).

### Outcome Variable

Data were captured on the six basic ADLs: walking across a room (mobility), bathing or showering, getting dressed, eating, getting into or out of sleeping location (transfers), and using the toilet (Katz et al., 1963). For the first ADL, walking across a room, participants were asked whether they had no difficulty, some difficulty, or were unable to perform the ADL. For the other ADLs, participants were asked whether they had no difficulty, mild difficulty, moderate difficulty, severe difficulty, extreme difficulty, were unable, or did not want to perform the ADL. As the latter answering mode depicts a broader range of difficulty an individual might have with an ADL, we harmonized responses to all six ADLs. We categorized some difficulty walking across a room with mild to moderate impairment group and inability to walk across a room with severe to extreme impairment. Respondents answering with “do not want to do” to the ADL questions were put in the severe to extreme impairment group, as the ADLs are essential activities to maintain an independent life and by reporting their unwillingness to engage in these pivotal activities, we considered them severely disabled. This harmonization is illustrated in Supplementary Figure 1.

Our primary bivariable and multivariable analyses use a binary measure for whether respondents reported any impairment on one or more ADLs. Additional analyses investigated whether relationships differed among individuals reporting mild to moderate impairment or severe to extreme impairment on one or more ADLs. These separate models have been conducted to show a more detailed picture of the subgroups composing people with ADL impairment. We present the findings of the binary logistic regression model given that this reflects the way that similar analyses have been presented before and therefore conveys the key results most effectively to readers while facilitating comparison with other studies.

If an individual reported any difficulty with an ADL, there was a follow-up question about whether the individual received from others: no help; some, but not enough help; or enough help for that ADL. We classified as follows: no help as an unmet need for care; some, but not enough help as a partially met need; and enough help as a met need. We then aggregated across ADLs to arrive at a summary measure. Those with any unmet need were classified as having “unmet need.” Those having no unmet need but reporting at least one partially met need were classified as having “partially met need.” Those with neither unmet nor partially met needs were classified as “met need.” We repeated the same process among the subgroup of respondents with severe to extreme ADL impairment to determine the rates of unmet need, partially met need, and met need among those most severely impaired.

### Explanatory Variables

We considered sociodemographic and socioeconomic characteristics commonly predictive of ADLs and care need. Age was segmented into 5-year groups to illustrate the prevalence of ADL impairment and care need and used as a continuous variable in bi- and multivariable regression. A wealth index was constructed using a previously described method (Brinkmann et al., 2020; see Supplementary Note 1 for methods used). Education was categorized as a binary variable, any versus no education, based on the low prevalence of postprimary education. Being married or cohabiting was combined to create a variable that represents the physical presence of a partner or a spouse in the household.

To investigate the associations of health characteristics with outcome variables, the Fried frailty score (Fried et al., 2001; Witham et al., 2019), a binary variable indicating the presence of cardiovascular disease (CVD) or cardiovascular disease risk factors (CVDRFs), a continuous measure of depressive symptoms based on the nine-item Patient Health Questionnaire depression module (Kroenke et al., 2010), and an abbreviated version of the Community Screening Instrument for Dementia (Hall et al., 1993) to measure cognitive impairment were used. A detailed description of how the explanatory variables were constructed can be found in Supplementary Note 2.

### Analysis

First, we described baseline characteristics of the sample, calculating confidence intervals (CIs) for binomial variables using Clopper–Pearson intervals. For significance testing, in order to compare continuous variables, the independent-samples *t* test was used for normally distributed variables and the Mann–Whitney *U* test was used for nonnormally distributed variables. Accounting for the complex study design, we adjusted for possible clustering effects at the village level. We did not adjust at the household level because in 95.3% of the sampled households only 1 person participated in the survey. Bivariable correlations between outcome and explanatory variables were calculated using Pearson’s correlation coefficients and chi-squared tests. Fisher *r*-to-*z* transformation was used to determine confidence intervals for Pearson’s *r*. We used logistic regression to calculate associations between explanatory variables and dependent variables. We included age, gender, wealth index, education, and married/cohabiting in all models. Additionally, we used ordinal logistic regression to account for the natural ordering of outcome categories; explanatory variables were, again, the above-mentioned sociodemographic variables. Analyses were conducted in SPSS v24 and 25 (IBM, Armonk, NY).

### Ethics

This study received ethics approval from the National Health Ethics Committee in Ouagadougou (#2018-5-053), the CRSN institutional ethics committee in Nouna (#2018-04), and the ethics committee of the medical faculty of the Ruprecht-Karls-Universität Heidelberg (#S-120/2018).

## Results

### Sample

Summary statistics for the sample used in the present analysis are presented in Table 1.

**Table 1.** Sample Characteristics

	Total (%)	Female (%)	Male (%)
	*N* = 3,026 (100)	*n* = 1,523 (50.3)	*n* = 1,503 (49.7)
Age mean (years)	54.3 (53.9–54.7)^a,^,*	55.5 (55.0–56.1)^a^	53.1 (52.6–53.6)^a^
Age groups (years)			
40–44	685 (22.6)*	300 (19.7)	385 (25.6)
45–49	579 (19.1)*	250 (16.4)	329 (21.9)
50–54	478 (15.8)	252 (16.5)	226 (15.0)
55–59	393 (13.0)	202 (13.3)	191 (12.7)
60–64	304 (10.0)*	178 (11.7)	126 (8.4)
65–69	249 (8.2)*	142 (9.3)	107 (7.1)
70–74	166 (5.5)	94 (6.2)	72 (4.8)
≥75	172 (5.7)*	105 (6.9)	67 (4.5)
Wealth quintiles			
1^b^	601 (19.9)*	346 (22.7)	255 (17.0)
2	600 (19.8)	302 (19.8)	298 (19.8)
3	605 (20.0)	285 (18.7)	320 (21.3)
4	614 (20.3)	301 (19.8)	313 (20.8)
5^c^	606 (20.0)	289 (19.0)	317 (21.1)
Any formal schooling	471 (15.6)*	142 (9.3)	329 (21.9)
Married or cohabiting	2288 (75.6)*	915 (60.1)	1373 (91.4)
Fried score			
Robust	1270 (42.0)	630 (41.4)	640 (42.6)
Prefrail	1323 (43.7)	647 (42.5)	676 (45.0)
Frail	212 (7.0)*	125 (8.2)	87 (5.8)
Unable to calculate	221 (7.3)	121 (7.9)	100 (6.7)
CVD/CVDRF^d^	911 (30.1)*	504 (34.3)	407 (27.9)
CSI-D^e^			
Normal	2811 (92.9)*	1371 (90.0)	1440 (95.8)
Possible dementia	176 (5.8)*	120 (7.9)	56 (3.7)
Probable dementia	39 (1.3)*	32 (2.1)	7 (0.5)

^a^95% confidence interval.

^b^Poorest.

^c^Wealthiest.

^d^Cardiovascular disease and cardiovascular disease risk factors.

^e^Community Screening Instrument for Dementia, an indicator for cognitive impairment. Normal: zero to two incorrect answers, possible dementia: three to four incorrect answers, and probable dementia: five and more incorrect answers.

*p < .05 between men and women.

Of the 3,996 sampled individuals, 3,026 (75.7%) participants were found, consented, and completed questions on all six ADL items and the follow-up questions and were included in the analysis. There were no substantial demographic differences between the sampled and participating individuals (Supplementary Table 1).

### ADL Impairment

At least one ADL impairment of any category was reported by 1,202/3,026 (39.7% [95% CI: 38.0%–41.5%]) of all respondents (Table 2).

**Table 2.** Distribution of ADL Limitations and Unmet Need for Care by Gender

	Total (%)	Female (%)	Male (%)
	*N* = 3,026	*n* = 1,523	*n* = 1,503
ADL impairment			
No impairment at all	1,824 (60.3)*	818 (53.7)	1,006 (66.9)
At least one mild to moderate impairment	1,000 (33.0)*	582 (38.2)	418 (27.8)
At least one severe to extreme impairment	202 (6.7)*	123 (8.1)	79 (5.3)
Met need for care	60 (29.7)*	39 (31.7)	21 (26.6)
Partially met need for care	55 (27.2)	30 (24.4)	25 (31.6)
Unmet need for care	87 (43.1)*	54 (43.9)	33 (41.8)
At least one impairment of any category	1,202 (39.7)*	705 (46.3)	497 (33.1)
Met need for care	179 (14.9)*	114 (16.2)	65 (13.1)
Partially met need for care	208 (17.3)	112 (15.9)	96 (19.3)
Unmet need for care	815 (67.8)*	479 (67.9)	336 (67.6)
ADL			
1. Walking across a room			
No difficulty	2,270 (75.0)*	1,052 (69.1)	1,218 (81.0)
Mild to moderate difficulty	679 (22.4)*	423 (27.8)	256 (17.0)
Severe to extreme difficulty	77 (2.5)*	48 (3.2)	29 (1.9)
Any difficulty	756 (25.0)*	471 (30.9)	285 (19.0)
Met need for care	162 (21.4)*	104 (22.1)	58 (20.4)
Partially met need for care	161 (21.3)	91 (19.3)	70 (24.6)
Unmet need for care	433 (57.3)*	276 (58.6)	157 (55.1)
2. Dressing			
No difficulty	2,534 (83.7)*	1,225 (80.4)	1,309 (87.1)
Mild to moderate difficulty	456 (15.1)*	280 (18.4)	176 (11.7)
Severe to extreme difficulty	36 (1.2)	18 (1.2)	18 (1.2)
Any difficulty	492 (16.3)*	298 (19.6)	194 (12.9)
Met need for care	94 (19.1)*	58 (19.5)	36 (18.6)
Partially met need for care	103 (20.9)	58 (19.5)	45 (23.2)
Unmet need for care	295 (60.0)*	182 (61.1)	113 (58.2)
3. Bathing/showering			
No difficulty	2,453 (81.1)*	1,174 (77.1)	1,279 (85.1)
Mild to moderate difficulty	503 (16.6)*	308 (20.2)	195 (13.0)
Severe to extreme difficulty	70 (2.3)	41 (2.7)	29 (1.9)
Any difficulty	573 (18.9)*	349 (22.9)	224 (14.9)
Met need for care	103 (18.0)*	65 (18.6)	38 (17.0)
Partially met need for care	114 (19.9)	64 (18.3)	50 (22.3)
Unmet need for care	356 (62.1)*	220 (63.0)	136 (60.7)
4. Eating			
No difficulty	2,481 (82.0)*	1,208 (79.3)	1,273 (84.7)
Mild to moderate difficulty	494 (16.3)*	288 (18.9)	206 (13.7)
Severe to extreme difficulty	51 (1.7)	27 (1.8)	24 (1.6)
Any difficulty	545 (18.0)*	315 (20.7)	230 (15.3)
Met need for care	77 (14.1)	47 (14.9)	30 (13.0)
Partially met need for care	117 (21.5)	65 (20.6)	52 (22.6)
Unmet need for care	351 (64.4)*	203 (64.4)	148 (64.3)
5. Getting in/out of the bed			
No difficulty	2,411 (79.7)*	1,158 (76.0)	1,253 (83.4)
Mild to moderate difficulty	554 (18.3)*	332 (21.8)	222 (14.8)
Severe to extreme difficulty	61 (2.0)	33 (2.2)	28 (1.9)
Any difficulty	615 (20.3)*	365 (24.0)	250 (16.6)
Met need for care	105 (17.1)*	66 (18.1)	39 (15.6)
Partially met need for care	142 (23.1)	75 (20.5)	67 (26.8)
Unmet need for care	368 (59.8)*	224 (61.4)	144 (57.6)
6. Using the toilet			
No difficulty	2,422 (80.0)*	1,160 (76.2)	1,262 (84.0)
Mild to moderate difficulty	517 (17.1)*	314 (20.6)	203 (13.5)
Severe to extreme difficulty	87 (2.9)	49 (3.2)	38 (2.5)
Any difficulty	604 (20.0)*	363 (23.8)	241 (16.0)
Met need for care	107 (17.7)*	67 (18.5)	40 (16.6)
Partially met need for care	129 (21.4)	75 (20.7)	54 (22.7)
Unmet need for care	368 (60.9)*	221 (60.9)	147 (61.0)

*Notes:* ADL = activities of daily living. This table depicts an item-by-item distribution of the answers to the ADL questions 1–6 by the harmonized definition in total and by gender as well as numbers and percentages of people with no impairment at all, people with at least one ADL impairment of any category, and people with at least one ADL impairment of the severe to extreme category listed in total and by gender. Additionally, numbers and percentages of met, partially met, and unmet need for care among those reporting any ADL difficulty and those reporting severe to extreme ADL difficulty are listed item-by-item as well as overall in total and by gender.

*p < .05 between men and women.

**Figure 1.**
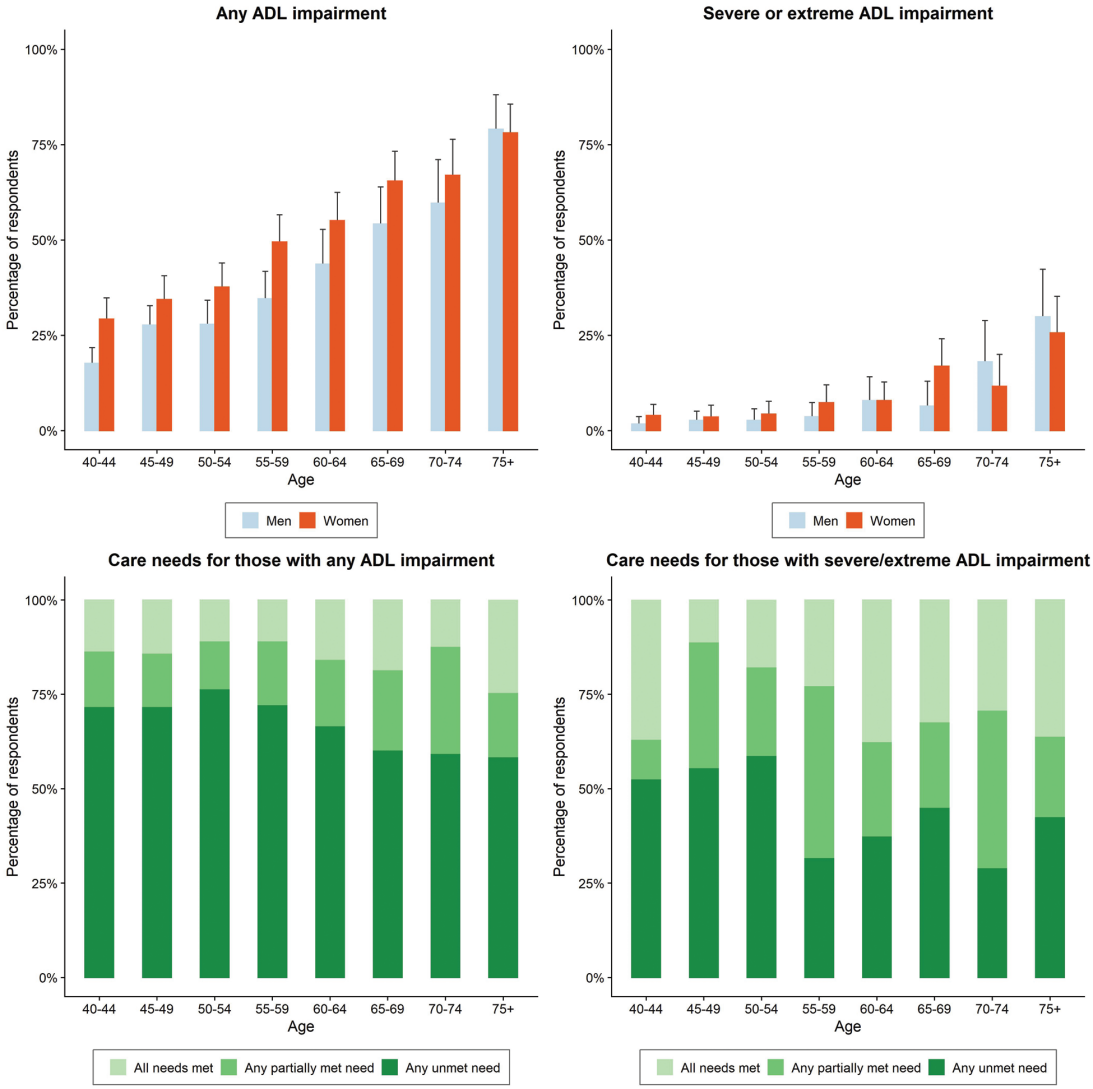
Prevalence of any activities of daily living (ADL) impairment and severe to extreme ADL impairment by age groups and sex; unmet, partially met, and met need by age groups.

Mild to moderate ADL impairment was reported by 1,000/3,026 (33.0% [31.4%–34.8%]) of all respondents and made up 83.2% (1,000/1,202) of those reporting an ADL impairment. Prevalence of reporting any ADL impairment increased with age, from 22.8% (19.7%–26.1%) of those aged 40–44 to 78.5% (71.6%–84.4%) of those aged older than 75 (Figure 1). Women had significantly higher rates of ADL impairment (46.3% [43.8%–48.8%], *p* < .001) than men (33.1% [30.7%–35.5%]). In bivariable, unadjusted analyses, which are presented in Supplementary Table 2, reporting any ADL impairment was positively associated with older age, being a woman, being frail, reporting a CVD/CVDRF, depressive symptoms, and possible as well as probable dementia. Respondents who were married or cohabiting and respondents who had any formal schooling as well as those who were categorized as “robust” on the Fried score had lower levels of ADL impairment in bivariable, unadjusted analyses. In multivariable regression analyses, older age (adjusted odds ratio [AOR]: 1.05 [1.04–1.06]), being a woman (AOR: 1.33 [1.06–1.60]), and reporting depressive symptoms (AOR: 1.90 [1.65–2.18]) were positively associated with ADL impairment of any type (Table 3). Additional analyses for the mild to moderate ADL impairment group did not show different results to analysis with all ADLs included (Supplementary Table 3).

**Table 3.** Multivariable Logistic Regressions Models Indicating Associations Between Sociodemographic and Health Characteristics and ADL Impairment as well as Unmet Need

	Any ADL impairment		Severe to extreme ADL impairment		Unmet need in those with any ADL impairment		Unmet need in those with severe to extreme ADL impairment	
	Model 1 (*n* = 3,026)		Model 2 (*n* = 3,026)		Model 3 (*n* = 1,202)		Model 4 (*n* = 202)	
Item	OR	95% CI	OR	95% CI	OR	95% CI	OR	95% CI
Age	1.05*	1.04–1.06	1.03*	1.01–1.05	0.99*	0.97–1.00	0.99	0.96–1.01
Gender^a^	1.33*	1.06–1.60	1.05	0.77–1.44	1.26	0.99–1.60	1.14	0.48–2.70
Wealth quintile								
1^b^	1	—	1	—	1	—	1	—
2	1.01	0.76–1.34	1.05	0.54–2.08	1.07	0.64–1.77	1.20	0.40–3.57
3	1.27	0.95–1.70	0.99	0.57–1.71	1.73*	1.02–2.93	1.46	0.61–3.47
4	1.38*	1.02–1.88	1.02	0.59–1.75	1.21	0.77–1.91	1.07	0.41–2.80
5^c^	1.48*	1.01–2.18	1.29	0.64–2.63	1.13	0.77–1.63	1.22	0.48–3.15
Any education^d^	1.06	0.83–1.37	0.80	0.49–1.30	1.01	0.68–1.49	1.68	0.63–4.52
Married/cohabiting^e^	0.89	0.69–1.14	0.94	0.60–1.48	1.45*	1.06–1.98	1.26	0.59–2.69
Fried score								
Robust	1	—	1	—	1	—	1	—
Prefrail	0.90	0.76–1.08	1.50	0.99–2.27	1.28	0.89–1.85	0.92	0.47–1.83
Frail	1.33	0.89–2.01	2.38*	1.31–4.32	1.29	0.78–2.14	0.67	0.27–1.66
Unable to calculate	1.06	0.75–1.49	2.67*	1.69–4.24	1.07	0.60–1.92	0.32	0.14–0.75
CVD/CVDRF^f^	1.03	0.86–1.22	1.27	0.87–1.86	0.87	0.62–1.22	1.00	0.44–2.26
PHQ-9^g^	1.90*	1.65–2.18	2.55*	2.11–3.07	0.78*	0.65–0.92	1.14	0.86–1.51]
CSI-D^h^								
Normal	1	—	1	—	1	—	1	—
Possible dementia	1.18	0.76–1.85	1.47	0.95–2.28	1.55	1.00–2.40	1.61	0.87–2.98]
Probable dementia	0.94	0.40–2.23	0.94	0.39–2.25	1.40	0.64–3.03	2.25	0.52–9.69

*Note:* ADL = activities of daily living; OR = odds ratio; CI = confidence interval.

^a^Reference: male.

^b^Poorest.

^c^Wealthiest.

^d^Reference: no formal education.

^e^Reference: not being married or cohabiting.

^f^Cardiovascular disease and cardiovascular disease risk factors.

^g^Patient Health Questionnaire 9, an indicator for depressive symptoms.

^h^Community Screening Instrument for Dementia, an indicator for cognitive impairment. Normal: zero to two incorrect answers, possible dementia: three to four incorrect answers, and probable dementia: five and more incorrect answers.

*p < .05.

At least one severe to extreme ADL impairment was reported by 6.7% (5.8%–7.6%) of all respondents (Table 2). Prevalence of severe to extreme ADL impairment increased from 2.8% (1.7%–4.3%) in the 40–44 age group to 27.3% (20.8%–34.6%) in the oldest age group. Women and older adults were most likely to be affected by severe to extreme ADL impairment. In bivariable analyses (Supplementary Table 2), severe to extreme ADL impairment was associated positively with being frail, reporting a CVD/CVDRF, depressive symptoms, and possible and probable dementia. Respondents who were married or cohabiting and formally educated or “robust” also had lower levels of severe to extreme ADL impairment in bivariable analyses. In multivariable analyses, severe to extreme ADL impairment was associated with older age (AOR: 1.03 [1.01–1.05]), a weaker association than with any ADL impairment. However, depressive symptoms (AOR: 2.55 [2.11–3.07]) were associated with severe to extreme ADL impairment to a stronger degree than with any ADL impairment. Additionally, being frail (AOR: 2.38 [1.31–4.32]) and being unable to complete the Fried score (AOR: 2.67 [1.69–4.24]) were associated strongly with severe to extreme ADL impairment. In additional analyses models run separately for men and women, it was found that the Fried score categories “frail” and “unable to score” were associated with men reporting severe to extreme ADL impairment, but not women (Supplementary Tables 3–5).

### Need for Care in People With ADL Impairment

When respondents reported any difficulty in performing an ADL, more unmet need than partially met or met need for care was reported for each of the six ADL items separately (Table 2). Having difficulty with “eating” caused the highest degree of unmet need (351/545 [64.4%]), while difficulty with “walking across a room” produced the lowest percentage (433/765 [57.3%]). Women reported more unmet need for care in the single items except “eating” and “getting in/out of the bed,” for which unmet need was equally distributed between men and women.

Of respondents with any ADL impairment, 67.8% (65.1%–70.4%) had at least one unmet need for care, 17.3% (15.2%–19.6%) had partially met need, and 14.9% (12.9%–17.0%) had all their ADL-related needs met. The proportion of unmet need was higher in younger age groups and declined with age (Figure 1). Among people aged between 50 and 54 with at least one ADL impairment of any category, 76.6% (69.2%–82.9%) reported at least one unmet need for care. This was significantly higher than the percent of people with unmet needs in age 65–69 (60.3% [52.0%–68.1%]), 70–74 (59.4% [49.5%–68.9%]), and 75 and older (58.5% [49.7%–66.9%]). In bivariable analyses, unmet need in those with any ADL impairment was associated with younger age, being married or cohabiting, and reporting fewer depressive symptoms (Supplementary Table 2). In multivariable analyses, depressive symptoms were negatively associated with unmet need (AOR: 0.78 [0.65–0.92]), while the other associations seen in unadjusted analyses diminished. Being married or cohabiting was associated positively with unmet need in women, but not in men (Supplementary Tables 6, 7 and 8).

In people with severe to extreme ADL impairment, the percentages of unmet need for care were significantly lower than in those with any ADL impairment (43.1% [36.1%–50.2%]). At least one partially met need, but no unmet need was reported by 27.2% [21.2%–33.9%]; and 29.7% [23.5%–36.5%] reported that all their ADL-related needs were met. At higher ages, people with severe to extreme ADL impairment more often reported partially met need and met need than unmet need (Figure 1) although these differences were not statistically significant.

When using ordinal logistic regression to account for the natural ordering of outcome categories, the results, presented in Supplementary Table 9, did not differ substantially from multivariable logistic regression.

## Discussion

Our analysis of a population-based representative sample of adults aged 40 years and older in a rural, poor area in Burkina Faso found that almost two out of five respondents had impaired ability to perform central ADLs. Most of these ADL limitations were mild to moderate, but one in 15 had severe or extreme ADL limitations. In a setting where care provision is largely informal and family-based (Aboderin, 2010), this places a substantial burden on households that are unlikely to be able to meet the care need of their relatives; two thirds of those with ADL impairment reported at least one unmet ADL-related need and a further one sixth a partially met need for care. These unmet needs are likely to have adverse effects on health and quality of life for those living with ADL limitations.

### ADL Impairment

The number of people with severe to extreme activity impairment found in our study (6.7%) is relatively low when compared to other studies conducted in Burkina Faso (Berthe et al., 2012, 2014; Mitra et al., 2013) that found one tenth to a third of their respondents to be activity-impaired or disabled, although only one study (Mitra et al., 2013) used the same age cutoff as ours. Conversely, the percentage of people with mild to moderate activity impairment in our sample (33.0%) was very high. It is possible that in Burkina Faso individuals with mild to moderate ADL limitations might die before they become severely impaired (Berthé et al., 2014), leading to a relatively low number of people with severe impairment. When adding up the categories, close to two fifths (39.7%) of the respondents in our study had some form of ADL impairment, which is one of the highest proportions ever measured in Burkina Faso. Nonetheless, differences in sample selection, categorization, and methodology make a comparison of studies about activity impairment within Burkina Faso difficult.

Older women had the highest burden of ADL impairment in our sample. This confirms results from previous studies in Burkina Faso (Miszkurka et al., 2012), Nigeria (Gureje et al., 2006), Cameroon (Kuate-Defo, 2005), and South Africa (Harling et al., 2020; Santosa et al., 2016). There may be several reasons for this differential burden by gender. First, gendered inequalities in educational opportunities and food insecurity during childhood have been posited as risk factors for Burkinabé women’s higher likelihood of developing ADL impairment later in life (Onadja et al., 2013). Second, multimorbidity, more common among older women in this sample (Odland et al., 2020), is linked to ADL impairment (Calderón-Larrañaga et al., 2019).

Third, although more conclusive evidence is necessary, biological differences between men and women at older ages may also contribute to the observed gender-specific patterns (Bird & Rieker, 2008). Specifically, the discrepancy in the health and survival of men and women has been well documented in other contexts, showing that men experience fewer disabilities throughout life but have higher mortality at all ages (Oksuzyan et al., 2008). In addition to differences in behavioral factors, these sex-specific differences in health outcomes have been attributed to genetic factors, differences in the immune system responses, and differential experience of diseases during the life course (Merz & Cheng, 2016). Prior research has also documented that at ages older than 65 years, women are characterized by lower functional capabilities than men, but the rate of aging is slower than that of men (Nakamura & Miyao, 2008).

Fourth, living with a spouse is known to protect against ADL impairment (Berthé et al., 2014; Sogebi et al., 2015); married women in Burkina Faso, as in much of SSA, are generally some years younger than their male spouses and have a longer life expectancy. This leads to a significantly higher number of female widows (in our sample, women comprised 88% of all widows). In our analyses, being married or cohabiting was associated with less ADL impairment in unadjusted analysis, although this association diminished after controlling for sociodemographic variables.

Berthé et al. (2014) argue that in the rural Burkina Faso setting, activity impairments in older age are experienced as something inevitable, normal. In our sample, ADL impairment of any category and severe to extreme ADL impairment were significantly associated with depressive symptoms in bivariable and multivariable analyses. Although causality cannot be established from our analysis, this association might reflect the adverse effects of ADL impairment on people’s mental health or the negative influence of depressive symptoms on the ability to perform everyday activities. However, adding to the association of depressive symptoms with CVD/CVDRF found in this sample previously (Brinkmann et al., 2020), these results show that people with depressive symptoms may be a very vulnerable group in this poor, rural context.

A positive association between people reporting any ADL impairment and wealth quintiles 4 and 5, indicating higher socioeconomic status, was found in multivariable analysis. As this association was relatively weak and other models did not show similar associations, we concluded that there was no clear pattern that would give us more insight into the understanding of ADL limitations and socioeconomic status in this context. In separate multivariate analyses for men and women, Fried categories “frail” and “unable to score” were positively associated with severe to extreme ADL impairment in men, but not in women. As the other independent variables showed no substantial differences, we assumed that this finding was due to chance.

### Unmet Need for Care

Previous work in Burkina Faso did not determine levels of unmet need for care among people with activity impairment. The figures of unmet need for care among activity-impaired persons (67.8%) were almost triple those seen in rural South Africa (Harling et al., 2020). They are also much higher than in rural and urban Nigeria, where one fifth of older than 65-year-old adults with ADL impairment did not have a caretaker available when needed (Gureje et al., 2006). The high prevalence of unmet need for care among people with activity impairment in this sample might partly be due to the paucity of formal care provision in the region of the study. Also, our survey instrument directly asked questions assessing unmet, partially met, and met need for care, allowing us to assess the perceived, rather than the objective, level of care need. Other reasons for the variance in the prevalence of unmet need might be differences in expectations of care receipt, differences in how the word “need” is interpreted in the respective cultures, differences in the perception of “need” in different countries, or differences in the amount of informal care available (e.g., family structures).

Among those with any ADL impairment, middle-aged people reported significantly higher rates of unmet need than older individuals. While older age has been found to be associated with unmet need in Nigeria (Gureje et al., 2006) and India (Singh et al., 2016), studies in Zimbabwe and South Africa revealed a similar reversed age gradient (Allain et al., 1997; Harling et al., 2020). Middle-aged people with ADL impairment possibly receive care from their parents, who might not be able to fulfill the individuals’ needs as well as it would be the other way around. Younger individuals with ADL impairment might be reluctant to ask for help as they struggle to accept their role as a person in need of help. On the other hand, the care need of younger people might sometimes be overlooked because it does not fit the stereotype of people in need of care (Harling et al., 2020).

Surprisingly, being married or cohabiting was positively associated with unmet need in multivariate analysis. At first sight, this finding does not align with results from other studies that have found more unmet needs in people who live without a spouse than in those sharing their household with a spouse (Harling et al., 2020; Singh et al., 2016). However, unmet need in this case might also be either the result of the limited informal caregivers’ ability to fulfill the need of the disabled person or a wish on the part of the care recipients to minimize the burden placed on carers by their care need (Choi & McDougall, 2009). Also, people may shape their thinking on whether or not their care need is being met in reference to whether there are available people around who could help. Individuals with a spouse may therefore be more likely to report unmet need, simply by virtue of having someone around who potentially could meet that need. Another hypothesis that needs further exploration is that people may be more concerned about those living alone and offer assistance to them, as they assume that individuals with a spouse are already helped.

In additional sensitivity analyses, the association between being married or cohabiting and reporting unmet need for care was statistically significant for women, but not for men (Supplementary Table 7). This finding possibly reflects gender-specific differences in the concept of illness and caregiving expectations. Men have been found to downplay illness experiences like ADL limitations, while women tend to experience illness more intensively (Umberson et al., 2016). Women seem to provide more care in a relationship and may have higher expectations of the level of care they receive from a spouse (Noël-Miller, 2010). Also, women are less likely to choose their spouse as a caregiver (Allen et al., 1999). They have to turn to other care structures, which may not be able to meet their care need. These mechanisms underlie great culture-specific differences, but women provide the majority of care also in African families and suffer from health problems like depression and anxiety related to caregiving more often than men (Bhan et al., 2020). While compensating a “care deficit,” African women are increasingly dependent on care themselves (Schatz & Seeley, 2015). Interventions to support the informal care network in Burkina Faso and SSA provided primarily by women are urgently needed.

Depressive symptoms were less common in individuals with unmet need than in those with partially met or met need, in contrast to the positive association seen in previous studies (Choi & McDougall, 2009; Gureje et al., 2006). Prior analyses have shown that depressive symptoms increase linearly with age in the same sample (Brinkmann et al., 2020) and are thus least prevalent at ages where unmet need is greatest, potentially leading to the negative association. Additionally, people with depressive symptoms in care may feel as if they are a burden to their caregiver, which is another possible explanation for the negative association (Choi & McDougall, 2009). Also, individuals with depressive symptoms are more likely to seek health care advice in general (Herrman et al., 2002), which might lead to a better care provision also for ADL-related needs and possibly raises awareness that health care in general is being provided. However, the negative association was relatively weak and should not be interpreted as a sign that people with depressive symptoms and ADL impairment are to be neglected. Notably, people with mental disorders are oftentimes ostracized and expelled by societies in Burkina Faso (Berthé et al., 2014) and may not have the informal care network of family and friends they need.

### Strengths and Limitations

Our study has the strengths of being large, population-based, and representative of local adults aged 40 years and older, providing reassurance that our findings are locally valid, potentially generalizing to other rural areas in Burkina Faso and the Sahel region. The age cutoff of 40 means our overall findings are not directly comparable to the typically older cutoffs for aging studies in HICs; however, given the life expectancy at birth of Burkinabé people (61.2 years [United Nations, Department of Economic and Social Affairs, & Population Division, 2019]), we believe that the respondents include relevantly old individuals within this setting.

There are, however, some important limitations to consider. First, the cross-sectional design of the study does not make it possible to draw temporal causal interpretations. Second, case numbers of ADL-impaired respondents with partially met and met need were too low to detect significant associations with explanatory variables in multivariable analysis. Third, our choices of measures are important to consider. The concept of ADL as a measure of disability is widely used in HICs as well as low- and middle-income countries and makes a comparison with the related literature possible. Our decision to present results for both “any ADL impairment,” which comprises all degrees of severity and “severe to extreme ADL impairment,” including only the most severely impaired, reflects the lack of consensus in categorizing ADL impairment. However, presenting both allows for comparison with a range of previous studies. It was necessary to align categories of severity between walking and the other ADL impairments as direct mapping of categories was not possible due to different answering options.

## Conclusions

We found a high prevalence of ADL impairment and of unmet need for care in a population-based sample of more than 3,000 older adults in a poor rural Burkina Faso setting. ADL impairment was particularly prevalent in older women, people with frailty, and people with depressive symptoms, highlighting key groups for future interventions. Importantly, unmet need was not reported mainly by those who were the most impaired, but by younger respondents with ADL impairment of any category, including those with mild or moderate impairment. Future research should focus on the investigation of the informal care system within families and peers for people with activity impairment to understand where and how care provision could help best.

## Supplementary Material

gbab041_suppl_Supplementary_Material
