# Supplementary material for: Impairment in Activities of Daily Living and Unmet Need for Care Among Older Adults: A Population-Based Study From Burkina Faso
Source: J Gerontol B Psychol Sci Soc Sci. 2021 Mar 14;76(9):1880–92. doi: 10.1093/geronb/gbab041 (PMC8557831; doi:10.1093/geronb/gbab041)
Supplement: gbab041_suppl_Supplementary_Material [file gbab041_suppl_supplementary_material.docx]

**SUPPLEMENTARY MATERIAL**

**Supplementary Note 1:**

The wealth index was constructed using polychoric principal components analysis (PCA) methods (Kolenikov & Angeles, 2009). This method is similar to the PCA method (Filmer & Pritchett, 2001) commonly used by Demographic and Household Surveys (Rutstein & Johnson, 2004), but incorporates continuous variables and a lack of ownership into wealth index quantification. Information on the ownership of some standard household asset variables (radio, television, landline phone, computer, refrigerator, table, bicycle, motorcycle/scooter, and car/van) were only collected for a subset of households and were therefore dropped from analysis in order to obtain a wealth index for the largest number of households possible. Variables included in the final calculation included: main source of water (non-protected sources combined), time to water source, toilet type (flush toilets combined), whether toilet is shared, cooking fuel, separate room for kitchen, number of bedrooms, number of cows, horses, goats, sheep, poultry, and other animals, hectares of land, electricity, chairs, dresser/bookshelf, watch, animal-pulled cart, cell phone, and bank account. The resulting wealth index (wealth_index) and wealth quintiles (wealth_quintile) are available for 2,957 unique households (cn_hh_echant) with 3,028 individuals (indiv_id).

In 57 households, there was more than one respondent per household. For these households, we only used the response from one respondent to calculate the wealth index for the household. If one of the respondents was the head of household, we automatically used that respondent. If there was more than one head of household, then we used the younger of the two heads of households (typically in this situation the other respondent was very senior). Finally, in one case neither of the respondents selected to answer the survey were identified as the head of household, in that case we selected the youngest respondent's answer.

**Supplementary Note 2:**

The Fried score was used to calculate frailty using the original five domains (weight loss, low grip strength, low walk speed, low activity levels, and exhaustion). The calculation described in Witham et al. (2019) was used.

We defined a binary variable indicating the presence of cardiovascular disease (CVD) or cardiovascular disease risk factors (CVDRF) when a respondent self-reported a diagnosis from a health care worker of at least one of the following conditions: elevated blood pressure; elevated blood sugar; abnormal cholesterol; heart disease; stroke or symptom suggestive of having had a stroke (sudden drooping of one side of the face; sudden numbness, weakness or dead feeling on one half of the body; sudden difficulty speaking or slurring of speech).

We used a continuous measure of depressive symptoms based on the 9-item Patient Health Questionnaire depression module (PHQ-9). The PHQ-9 is an instrument designed for primary care, either to make a probable diagnosis of major depressive disorder (MDD) or to measure depressive symptoms longitudinally, with a possible range of 0 to 27 (Kroenke, Spitzer, Williams, & Lowe, 2010). Since responses varied systematically by interviewer, we calculated z-scores at the interviewer level (Brinkmann et al., 2020).

Lastly, we used an abbreviated version of the Community Screening Instrument for Dementia (CSI-D), which has been widely used and validated to measure dementia symptoms in low- and middle-income countries (Guerchet et al., 2014), to measure cognitive impairment (Hall et al., 1993). Respondents answering incorrectly to 0 to 2 of the 9 items were categorized as ‘normal’. 3 to 4 incorrect answers meant to be categorized as having ‘possible dementia’, 5 or more incorrect answers as having ‘probable dementia’ (Prince et al., 2011).

n = 3

n = 6

(1) Walking across a room

(2) Bathing or showering

(3) Getting dressed

(4) Eating

(5) Bed transferring

(6) Using the toilet

No difficulty

Some difficulty

Cannot do but want to do

Cannot do but do not need to do

No difficulty

Mild difficulty

Moderate difficulty

Severe difficulty

Extreme difficulty or cannot do

Do not want to do

No difficulty

Mild to moderate difficulty

Severe to extreme difficulty

No difficulty

Mild to moderate difficulty

Severe to extreme difficulty

Answering option

Harmonized categorization

Item

**Supplementary Figure 1:** Definition of ADL difficulty for (1) walking and harmonization of the other ADLs (2-6). n = 6 respondents did not feel the need to perform ADL 1 (walking). n = 1 respondent did not want to perform ADL 2 (bathing), n = 0 respondents did not want to perform ADL 3 (dressing), n = 1 respondents did not want to perform ADL 4 (eating), n = 0 respondents did not want to perform ADL 5 (transferring) and n = 1 respondents did not want to perform ADL 6 (toileting). This makes a total of n = 3 respondents who did not want to perform ADLs 2-6.

| **Supplementary Table 1:** Gender distribution and age means of the non-participating, sampled population | | | |
| --- | --- | --- | --- |
|  | **Total (%)** | **Female (%)** | **Male (%)** |
|  | ***N* = 852^a^** | ***n* = 444 (52.1)** | ***n* = 408 (47.9)** |
| **Age mean (y)^b^** | 56.2 [95% CI: 55.2-57.8] | 58.8 [95% CI: 57.4-60.2] | 53.8 [95% CI: 52.5-55.1] |
| ^a^ 118 missing values. ^b^114 missing values | | | |

| **Supplementary Table 2:** Bivariate, unadjusted correlations of sociodemographic and health characteristics, any ADL impairment, severe to extreme ADL impairment, as well as unmet need in those with any ADL impairment and severe to extreme ADL impairment. | | | | | | | | |
| --- | --- | --- | --- | --- | --- | --- | --- | --- |
|  | **Any ADL^a^ impairment** | | **Severe to extreme ADL^a^ impairment** | | **Unmet need in those with any ADL^a^ impairment** | | **Unmet need in those with severe to extreme ADL^a^ impairment** | |
|  | Model 1 (n=3026) | | Model 2 (n=3026) | | Model 3 (n=1202) | | Model 4 (n=202) | |
| Item | UOR^b^ | 95% CI^c^ | UOR^b^ | 95% CI^c^ | UOR^b^ | 95% CI^c^ | UOR^b^ | 95% CI^c^ |
| Age | 1.07* | [1.06-1.08] | 1.07* | [1.06-1.09] | 0.98* | [0.97-0.99] | 1.02 | [0.99-1.04] |
| Gender^d^ | 1.75* | [1.43-2.12] | 1.58* | [1.21-2.08] | 1.02 | [0.81-1.27] | 1.09 | [0.54-2.20] |
| Wealth quintile |  |  |  |  |  |  |  |  |
| 1^e^ | 1.18 | [0.92-1.51] | 1.60 | [1.01-2.54] | 0.71 | [0.49-1.02] | 0.81 | [0.39-1.66] |
| 2 | 0.80* | [0.66-0.97] | 0.90 | [0.57-1.41] | 0.89 | [0.62-1.28] | 1.01 | [0.41-2.51] |
| 3 | 0.96 | [0.79-1.17] | 0.83 | [0.56-1.28] | 1.68* | [1.13-2.48] | 1.31 | [0.68-2.51] |
| 4 | 1.02 | [0.83-1.24] | 0.78 | [0.55-1.11] | 1.08 | [0.75-1.54] | 0.91 | [0.39-2.14] |
| 5^f^ | 1.07 | [0.82-1.41] | 0.99 | [0.65-1.48] | 0.94 | [0.71-1.24] | 1.10 | [0.51-2.37] |
| Any education^g^ | 0.78 | [0.60-1.02] | 0.58* | [0.39-0.86] | 1.13 | [0.79-1.63] | 1.70 | [0.66-4.39] |
| Married/cohabiting^h^ | 0.41* | [0.33-0.51] | 0.37* | [0.26-0.52] | 1.61* | [1.27-2.03] | 1.36 | [0.77-2.42] |
| Fried score |  |  |  |  |  |  |  |  |
| Robust^i^ | 0.60* | [0.51-0.70] | 0.26* | [0.18-0.39] | 1.09 | [0.77-1.54] | 1.40 | [0.71-2.78] |
| Pre-frail | 1.01 | [0.87-1.18] | 0.91 | [0.67-1.24] | 1.19 | [0.89-1.59] | 1.37 | [0.89-2.18] |
| Frail | 3.77* | [2.68-5.31] | 4.72* | [3.09-7.21] | 0.77 | [0.53-1.13] | 0.91 | [0.48-1.72] |
| Unable to calculate^j^ | 1.59* | [1.20-2.09] | 3.34* | [2.38-4.71] | 0.70 | [0.43-1.13] | 0.47 | [0.20-1.10] |
| CVD/CVDRF^k^ | 1.45* | [1.26-1.67] | 1.92* | [1.36-2.71] | 0.82 | [0.61-1.10] | 1.06 | [0.54-2.08] |
| PHQ9^l^ | 2.23* | [1.97-2.52] | 3.15* | [2.64-3.76] | 0.75* | [0.64-0.87] | 1.02 | [0.80-1.30] |
| CSI-D^m^ |  |  |  |  |  |  |  |  |
| Normal^n^ | 0.37* | [0.28-0.49] | 0.22* | [0.15-0.31] | 1.03 | [0.74-1.45] | 0.78 | [0.45-1.36] |
| Possible dementia | 2.31* | [1.63-3.25] | 3.50* | [2.44-5.04] | 1.13 | [0.74-1.73] | 1.20 | [0.67-2.16] |
| Probable dementia^o^ | 5.16* | [2.36-11.32] | 8.34* | [4.04-17.19] | 0.61 | [0.31-1.23] | 1.35 | [0.44-4.11] |
| *p<0.05  ^a^ Activities of Daily Living. ^b^ Unadjusted Odds Ratio. ^c^ confidence interval. ^d^ reference: male. ^e^ poorest, reference: quintiles 2-5. ^f^ wealthiest, reference: quintiles 1-4. Each category was compared to the remaining categories. ^g^ reference: no formal education. ^h^ reference: not being married or cohabiting. ^i^ reference: pre-frail, frail, unable to calculate. ^j^ reference: robust, pre-frail, frail. ^j^ Each category was compared to the remaining categories. ^k^ cardiovascular disease and cardiovascular disease risk factors. ^l^ Patient Health Questionnaire 9, indicator for depressive symptoms. ^m^ Community Screening Instrument for Dementia, indicator for cognitive impairment. Normal: 0-2 incorrect answers, possible dementia: 3-4 incorrect answers, probable dementia: 5+ incorrect answers. ^n^ reference: possible and probable dementia. ^o^ reference: normal, possible dementia. | | | | | | | | |

| **Supplementary Table 3:** Multivariable logistic regressions models indicating associations between sociodemographic and health characteristics and Mild to Moderate ADL impairment in total and, in separate models, for men and women. | | | | | | |
| --- | --- | --- | --- | --- | --- | --- |
|  | **Mild to Moderate ADL impairment (men and women)** | | **Mild to Moderate ADL impairment (men)** | | **Mild to Moderate ADL impairment (women)** | |
|  | Model 1 (n=3,026) | | Model 2 (n=1,503) | | Model 3 (n=1,523) | |
| Item | OR^b^ | 95% CI^c^ | OR^b^ | 95% CI^c^ | OR^b^ | 95% CI^c^ |
| Age | 1.04* | [1.02-1.05] | 1.04* | [1.03-1.06] | 1.03* | [1.01-1.05] |
| Gender^d^ | 1.40* | [1.16-1.68] | - | - | - | - |
| Wealth quintile |  |  |  |  |  |  |
| 1^e^ | 1 | - | 1 | - | 1 | - |
| 2 | 1.01 | [0.75-1.36] | 1.21 | [0.79-1.86] | 0.89 | [0.61-1.34] |
| 3 | 1.24 | [0.95-1.61] | 1.55* | [1.03-2.34] | 1.07 | [0.75-1.52] |
| 4 | 1.34* | [1.03-1.74] | 1.55 | [0.99-2.40] | 1.22 | [0.88-1.70] |
| 5^f^ | 1.36 | [1.00-1.84] | 1.86* | [1.15-3.00] | 1.06 | [0.71-1.56] |
| Any education^g^ | 1.11 | [0.87-1.41] | 0.94 | [0.67-1.32] | 1.46 | [0.91-2.34] |
| Married/cohabiting^h^ | 0.90 | [0.70-1.17] | 0.96 | [0.56-1.64] | 0.86 | [0.66-1.13] |
| Fried score |  |  |  |  |  |  |
| Robust | 1 | - | 1 | - | 1 | - |
| Pre-frail | 0.90 | [0.76-1.05] | 0.74* | [0.56-0.98] | 1.05 | [0.81-1.37] |
| Frail | 0.92 | [0.63-1.33] | 0.82 | [0.50-1.34] | 1.02 | [0.61-1.73] |
| Unable to calculate | 0.72 | [0.50-1.02] | 0.56 | [0.31-1.01] | 0.88 | [0.56-1.39] |
| CVD/CVDRF^i^ | 0.99 | [0.83-1.18] | 0.92 | [0.72-1.17] | 1.04 | [0.78-1.39] |
| PHQ9^j^ | 1.31* | [1.16-1.48] | 1.41* | [1.22-1.62] | 1.23* | [1.04-1.47] |
| CSI-D^k^ |  |  |  |  |  |  |
| Normal | 1 | - | 1 | - | 1 | - |
| Possible dementia | 0.89 | [0.58-1.39] | 1.05 | [0.48-2.31] | 0.86 | [0.54-1.38] |
| Probable dementia | 0.50 | [0.21-1.16] | 0.13 | [0.01-1.32] | 0.68 | [0.26-1.76] |
| *p<0.05  ^a^ Activities of Daily Living. ^b^ Odds Ratio. ^c^ confidence interval. ^d^ reference: male. ^e^ poorest. ^f^ wealthiest. ^g^ reference: no formal education. ^h^ reference: not being married or cohabiting. ^i^ cardiovascular disease and cardiovascular disease risk factors. ^j^ Patient Health Questionnaire 9, indicator for depressive symptoms. ^k^ Community Screening Instrument for Dementia, indicator for cognitive impairment. Normal: 0-2 incorrect answers, possible dementia: 3-4 incorrect answers, probable dementia: 5+ incorrect answers. | | | | | | |

| **Supplementary Table 4:** Multivariable logistic regressions models indicating associations between sociodemographic and health characteristics and Any ADL impairment in total and, in separate models, for men and women. | | | | | | |
| --- | --- | --- | --- | --- | --- | --- |
|  | **Any ADL impairment (men and women)** | | **Any ADL impairment (men)** | | **Any ADL impairment (women)** | |
|  | Model 1 (n=3,026) | | Model 2 (n=1,503) | | Model 3 (n=1,523) | |
| Item | OR^b^ | 95% CI^c^ | OR^b^ | 95% CI^c^ | OR^b^ | 95% CI^c^ |
| Age | 1.05* | [1.04-1.06] | 1.06* | [1.04-1.07] | 1.04* | [1.03-1.06] |
| Gender^d^ | 1.33* | [1.06-1.60] | - | - | - | - |
| Wealth quintile |  |  |  |  |  |  |
| 1^e^ | 1 | - | 1 | - | 1 | - |
| 2 | 1.01 | [0.76-1.34] | 1.31 | [0.81-2.11] | 0.86 | [0.61-1.21] |
| 3 | 1.27 | [0.95-1.70] | 1.64* | [1.07-2.51] | 1.07 | [0.72-1.58] |
| 4 | 1.38* | [1.02-1.88] | 1.77* | [1.10-2.86] | 1.17 | [0.79-1.73] |
| 5^f^ | 1.48* | [1.01-2.18] | 1.98* | [1.20-3.27] | 1.20 | [0.73-1.99] |
| Any education^g^ | 1.06 | [0.83-1.37] | 0.92 | [0.61-1.36] | 1.36 | [0.89-2.06] |
| Married/cohabiting^h^ | 0.89 | [0.69-1.14] | 0.99 | [0.61-1.61] | 0.83 | [0.62-1.10] |
| Fried score |  |  |  |  |  |  |
| Robust | 1 | - | 1 | - | 1 | - |
| Pre-frail | 0.90 | [0.76-1.08] | 0.77 | [0.58-1.03] | 1.04 | [0.79-1.38] |
| Frail | 1.33 | [0.89-2.01] | 1.32 | [0.74-2.34] | 1.36 | [0.81-2.30] |
| Unable to calculate | 1.06 | [0.75-1.49] | 1.00 | [0.60-1.65] | 1.14 | [0.76-1.71] |
| CVD/CVDRF^i^ | 1.03 | [0.86-1.22] | 0.95 | [0.72-1.25] | 1.08 | [0.81-1.44] |
| PHQ9^j^ | 1.90* | [1.65-2.18] | 2.05* | [1.78-2.36] | 1.77* | [1.48-2.14] |
| CSI-D^k^ |  |  |  |  |  |  |
| Normal | 1 | - | 1 | - | 1 | - |
| Possible dementia | 1.18 | [0.76-1.85] | 1.62 | [0.81-3.27] | 1.09 | [0.65-1.81] |
| Probable dementia | 0.94 | [0.40-2.23] | 0.41 | [0.08-2.01] | 1.20 | [0.45-3.22] |
| *p<0.05  ^a^ Activities of Daily Living. ^b^ Odds Ratio. ^c^ confidence interval. ^d^ reference: male. ^e^ poorest. ^f^ wealthiest. ^g^ reference: no formal education. ^h^ reference: not being married or cohabiting. ^i^ cardiovascular disease and cardiovascular disease risk factors. ^j^ Patient Health Questionnaire 9, indicator for depressive symptoms. ^k^ Community Screening Instrument for Dementia, indicator for cognitive impairment. Normal: 0-2 incorrect answers, possible dementia: 3-4 incorrect answers, probable dementia: 5+ incorrect answers. | | | | | | |

| **Supplementary Table 5:** Multivariable logistic regressions models indicating associations between sociodemographic and health characteristics and Severe to Extreme ADL impairment in total and, in separate models, for men and women. | | | | | | |
| --- | --- | --- | --- | --- | --- | --- |
|  | **Severe to Extreme ADL impairment (men and women)** | | **Severe to Extreme ADL impairment (men)** | | **Severe to Extreme ADL impairment (women)** | |
|  | Model 1 (n=3,026) | | Model 2 (n=1,503) | | Model 3 (n=1,523) | |
| Item | OR^b^ | 95% CI^c^ | OR^b^ | 95% CI^c^ | OR^b^ | 95% CI^c^ |
| Age | 1.03* | [1.01-1.05] | 1.03 | [1.00-1.06] | 1.02* | [1.00-1.05] |
| Gender^d^ | 1.05 | [0.77-1.44] | - | - | - | - |
| Wealth quintile |  |  |  |  |  |  |
| 1^e^ | 1 | - | 1 | - | 1 | - |
| 2 | 1.05 | [0.54-2.08] | 1.57 | [0.57-4.35] | 0.91 | [0.41-2.04] |
| 3 | 0.99 | [0.57-1.71] | 1.11 | [0.43-2.87] | 0.99 | [0.50-1.98] |
| 4 | 1.02 | [0.59-1.75] | 1.76 | [0.73-4.24] | 0.75 | [0.42-1.35] |
| 5^f^ | 1.29 | [0.64-2.63] | 1.27 | [0.46-3.49] | 1.40 | [0.67-2.95] |
| Any education^g^ | 0.80 | [0.49-1.30] | 0.87 | [0.47-1.62] | 0.68 | [0.24-1.93] |
| Married/cohabiting^h^ | 0.94 | [0.60-1.48] | 1.26 | [0.48-3.30] | 0.78 | [0.49-1.23] |
| Fried score |  |  |  |  |  |  |
| Robust | 1 | - | 1 | - | 1 | - |
| Pre-frail | 1.50 | [0.99-2.27] | 2.93* | [1.16-7.38] | 1.16 | [0.74-1.83] |
| Frail | 2.38* | [1.31-4.32] | 5.73* | [1.75-18.73] | 1.66 | [0.87-3.16] |
| Unable to calculate | 2.67* | [1.69-4.24] | 6.61* | [2.78-15.72] | 1.71 | [0.94-3.10] |
| CVD/CVDRF^i^ | 1.27 | [0.87-1.86] | 1.36 | [0.71-2.64] | 1.25 | [0.76-2.05] |
| PHQ9^j^ | 2.55* | [2.11-3.07] | 3.03* | [2.40-3.84] | 2.27* | [1.82-2.83] |
| CSI-D^k^ |  |  |  |  |  |  |
| Normal | 1 | - | 1 | - | 1 | - |
| Possible dementia | 1.47 | [0.95-2.28] | 1.89 | [0.78-4.61] | 1.42 | [0.85-2.39] |
| Probable dementia | 0.94 | [0.39-2.25] | 1.27 | [0.19-8.64] | 0.94 | [0.37-2.44] |
| *p<0.05  ^a^ Activities of Daily Living. ^b^ Odds Ratio. ^c^ confidence interval. ^d^ reference: male. ^e^ poorest. ^f^ wealthiest. ^g^ reference: no formal education. ^h^ reference: not being married or cohabiting. ^i^ cardiovascular disease and cardiovascular disease risk factors. ^j^ Patient Health Questionnaire 9, indicator for depressive symptoms. ^k^ Community Screening Instrument for Dementia, indicator for cognitive impairment. Normal: 0-2 incorrect answers, possible dementia: 3-4 incorrect answers, probable dementia: 5+ incorrect answers. | | | | | | |

| **Supplementary Table 6:** Multivariable logistic regressions models indicating associations between sociodemographic and health characteristics and unmet need for care in those with Mild to Moderate ADL impairment in total and, in separate models, for men and women. | | | | | | |
| --- | --- | --- | --- | --- | --- | --- |
|  | **Unmet need in Mild to Moderate ADL impairment (men and women)** | | **Unmet need in Mild to Moderate ADL impairment (men)** | | **Unmet need in Mild to Moderate ADL impairment (women)** | |
|  | Model 1 (n=1,000) | | Model 2 (n=418) | | Model 3 (n=582) | |
| Item | OR^b^ | 95% CI^c^ | OR^b^ | 95% CI^c^ | OR^b^ | 95% CI^c^ |
| Age | 0.99 | [0.97-1.00] | 0.99 | [0.96-1.01] | 0.98 | [0.96-1.00] |
| Gender^d^ | 1.26 | [0.93-1.70] | - | - | - | - |
| Wealth quintile |  |  |  |  |  |  |
| 1^e^ | 1 | - | 1 | - | 1 | - |
| 2 | 1.06 | [0.63-1.79] | 1.16 | [0.60-2.27] | 1.00 | [0.49-2.04] |
| 3 | 1.85* | [1.03-3.34] | 2.28 | [0.87-5.96] | 1.70 | [0.87-3.33] |
| 4 | 1.21 | [0.72-2.04] | 1.15 | [0.53-2.59] | 1.29 | [0.72-2.31] |
| 5^f^ | 1.13 | [0.70-1.83] | 1.23 | [0.53-2.82] | 1.08 | [0.63-1.85] |
| Any education^g^ | 0.86 | [0.55-1.36] | 1.45 | [0.72-3.10] | 0.45* | [0.26-0.80] |
| Married/cohabiting^h^ | 1.46* | [1.00-2.14] | 1.38 | [0.66-2.89] | 1.42 | [0.97-2.08] |
| Fried score |  |  |  |  |  |  |
| Robust | 1 | - | 1 | - | 1 | - |
| Pre-frail | 1.42 | [0.93-2.15] | 1.56 | [0.86-2.84] | 1.30 | [0.78-2.16] |
| Frail | 1.77 | [0.97-3.22] | 2.20 | [0.72-6.80] | 1.63 | [0.78-3.42] |
| Unable to calculate | 1.84 | [0.91-3.75] | 1.58 | [0.65-3.87] | 1.90 | [0.85-4.23] |
| CVD/CVDRF^i^ | 0.88 | [0.61-1.27] | 0.74 | [0.44-1.27] | 1.07 | [0.67-1.70] |
| PHQ9^j^ | 0.81* | [0.67-0.97] | 0.82 | [0.65-1.04] | 0.80 | [0.63-1.02] |
| CSI-D^k^ |  |  |  |  |  |  |
| Normal | 1 | - | -^l^ | -^l^ | 1 | - |
| Possible dementia | 1.81 | [0.95-3.45] | -^l^ | -^l^ | 1.97 | [0.84-4.65] |
| Probable dementia | 0.92 | [0.29-2.95] | -^l^ | -^l^ | 0.80 | [0.24-2.62] |
| *p<0.05  ^a^ Activities of Daily Living. ^b^ Odds Ratio. ^c^ confidence interval. ^d^ reference: male. ^e^ poorest. ^f^ wealthiest. ^g^ reference: no formal education. ^h^ reference: not being married or cohabiting. ^i^ cardiovascular disease and cardiovascular disease risk factors. ^j^ Patient Health Questionnaire 9, indicator for depressive symptoms. ^k^ Community Screening Instrument for Dementia, indicator for cognitive impairment. Normal: 0-2 incorrect answers, possible dementia: 3-4 incorrect answers, probable dementia: 5+ incorrect answers. ^l^ Not enough cases to compute ORs (only one male individual with probable dementia and mild to moderate ADL impairment). | | | | | | |

| **Supplementary Table 7:** Multivariable logistic regressions models indicating associations between sociodemographic and health characteristics and unmet need for care in those with Any ADL impairment in total and, in separate models, for men and women. | | | | | | |
| --- | --- | --- | --- | --- | --- | --- |
|  | **Unmet need in Any ADL impairment (men and women)** | | **Unmet need in Any ADL impairment (men)** | | **Unmet need in Any ADL impairment (women)** | |
|  | Model 1 (n=1,202) | | Model 2 (n=497) | | Model 3 (n=705) | |
| Item | OR^b^ | 95% CI^c^ | OR^b^ | 95% CI^c^ | OR^b^ | 95% CI^c^ |
| Age | 0.99* | [0.97-1.00] | 0.99 | [0.96-1.01] | 0.98 | [0.97-1.00] |
| Gender^d^ | 1.26 | [0.99-1.60] | - | - | - | - |
| Wealth quintile |  |  |  |  |  |  |
| 1^e^ | 1 | - | 1 | - | 1 | - |
| 2 | 1.07 | [0.64-1.77] | 0.90 | [0.49-1.67] | 1.18 | [0.61-2.28] |
| 3 | 1.73* | [1.02-2.93] | 2.02 | [0.96-4.23] | 1.59 | [0.82-3.10] |
| 4 | 1.21 | [0.77-1.91] | 1.03 | [0.54-1.98] | 1.38 | [0.80-2.38] |
| 5^f^ | 1.13 | [0.77-1.63] | 1.17 | [0.60-2.28] | 1.09 | [0.70-1.68] |
| Any education^g^ | 1.01 | [0.68-1.49] | 1.40 | [0.79-2.49] | 0.67 | [0.38-1.16] |
| Married/cohabiting^h^ | 1.45* | [1.06-1.98] | 1.29 | [0.70-2.38] | 1.48* | [1.05-2.08] |
| Fried score |  |  |  |  |  |  |
| Robust | 1 | - | 1 | - | 1 | - |
| Pre-frail | 1.28 | [0.89-1.85] | 1.34 | [0.78-2.29] | 1.24 | [0.80-1.92] |
| Frail | 1.29 | [0.78-2.14] | 1.63 | [0.72-3.69] | 1.12 | [0.57-2.19] |
| Unable to calculate | 1.07 | [0.60-1.92] | 0.69 | [0.34-1.40] | 1.40 | [0.66-2.98] |
| CVD/CVDRF^i^ | 0.87 | [0.62-1.22] | 0.82 | [0.52-1.28] | 0.93 | [0.62-1.40] |
| PHQ9^j^ | 0.78* | [0.65-0.92] | 0.78* | [0.63-0.96] | 0.78 | [0.61-1.00] |
| CSI-D^k^ |  |  |  |  |  |  |
| Normal | 1 | - | 1 | - | 1 | - |
| Possible dementia | 1.55 | [1.00-2.40] | 1.18 | [0.48-2.92] | 1.75 | [0.98-3.11] |
| Probable dementia | 1.40 | [0.64-3.03] | 2.38 | [0.40-14.31] | 1.31 | [0.54-3.17] |
| *p<0.05  ^a^ Activities of Daily Living. ^b^ Odds Ratio. ^c^ confidence interval. ^d^ reference: male. ^e^ poorest. ^f^ wealthiest. ^g^ reference: no formal education. ^h^ reference: not being married or cohabiting. ^i^ cardiovascular disease and cardiovascular disease risk factors. ^j^ Patient Health Questionnaire 9, indicator for depressive symptoms. ^k^ Community Screening Instrument for Dementia, indicator for cognitive impairment. Normal: 0-2 incorrect answers, possible dementia: 3-4 incorrect answers, probable dementia: 5+ incorrect answers. | | | | | | |

| **Supplementary Table 8:** Multivariable logistic regressions models indicating associations between sociodemographic and health characteristics and unmet need for care in those with Severe to Extreme ADL impairment in total and, in separate models, for men and women. | | | | | | |
| --- | --- | --- | --- | --- | --- | --- |
|  | **Unmet need in Severe to Extreme ADL impairment (men and women)** | | **Unmet need in Severe to Extreme ADL impairment (men)** | | **Unmet need in Severe to Extreme ADL impairment (women)** | |
|  | Model 1 (n=202) | | Model 2 (n=79) | | Model 3 (n=123) | |
| Item | OR^b^ | 95% CI^c^ | OR^b^ | 95% CI^c^ | OR^b^ | 95% CI^c^ |
| Age | 0.99 | [0.96-1.01] | 0.98 | [0.94-1.02] | 0.99 | [0.95-1.03] |
| Gender^d^ | 1.14 | [0.48-2.70] | - | - | - | - |
| Wealth quintile |  |  |  |  |  |  |
| 1^e^ | 1 | - | 1 | - | 1 | - |
| 2 | 1.20 | [0.40-3.57] | 0.38 | [0.08-1.79] | 2.26 | [0.67-7.64] |
| 3 | 1.46 | [0.61-3.47] | 1.88 | [0.33-10.61] | 1.77 | [0.50-6.23] |
| 4 | 1.07 | [0.41-2.80] | 0.79 | [0.13-4.97] | 1.40 | [0.45-4.30] |
| 5^f^ | 1.22 | [0.48-3.15] | 1.18 | [0.20-7.11] | 1.61 | [0.48-5.38] |
| Any education^g^ | 1.68 | [0.63-4.52] | 0.68 | [0.19-2.42] | 3.60 | [0.64-20.23] |
| Married/cohabiting^h^ | 1.26 | [0.59-2.69] | 1.12 | [0.17-7.31] | 1.42 | [0.63-3.20] |
| Fried score |  |  |  |  |  |  |
| Robust | 1 | - | 1 | - | 1 | - |
| Pre-frail | 0.92 | [0.47-1.83] | 0.78 | [0.16-3.85] | 1.06 | [0.49-2.30] |
| Frail | 0.67 | [0.27-1.66] | 1.04 | [0.17-6.38] | 0.59 | [0.15-2.26] |
| Unable to calculate | 0.32* | [0.14-0.75] | 0.11* | [0.02-0.64] | 0.58 | [0.18-1.92] |
| CVD/CVDRF^i^ | 1.00 | [0.44-2.26] | 1.85 | [0.53-6.43] | 0.73 | [0.31-1.74] |
| PHQ9^j^ | 1.14 | [0.86-1.51] | 1.30 | [0.79-2.14] | 1.08 | [0.75-1.55] |
| CSI-D^k^ |  |  |  |  |  |  |
| Normal | 1 | - | 1 | - | 1 | - |
| Possible dementia | 1.61 | [0.87-2.98] | 0.85 | [0.14-5.04] | 2.29* | [1.03-5.12] |
| Probable dementia | 2.25 | [0.52-9.69] | 1.64 | [0.12-21.76] | 2.70 | [0.46-16.03] |
| *p<0.05  ^a^ Activities of Daily Living. ^b^ Odds Ratio. ^c^ confidence interval. ^d^ reference: male. ^e^ poorest. ^f^ wealthiest. ^g^ reference: no formal education. ^h^ reference: not being married or cohabiting. ^i^ cardiovascular disease and cardiovascular disease risk factors. ^j^ Patient Health Questionnaire 9, indicator for depressive symptoms. ^k^ Community Screening Instrument for Dementia, indicator for cognitive impairment. Normal: 0-2 incorrect answers, possible dementia: 3-4 incorrect answers, probable dementia: 5+ incorrect answers. | | | | | | |

| **Supplementary Table 9:** Ordinal logistic regression models | | | | |
| --- | --- | --- | --- | --- |
|  | Dependent variable:  **ADL impairment**  **None /**  **Mild to Moderate /**  **Severe to Extreme** | | Dependent variable:  **Need for care**  **All needs met /**  **Any partially met need /**  **Any unmet need** | |
|  | Model 1 (n=3026) | | Model 2 (n=3026) | |
|  | OR^a^ | 95% CI^b^ | OR^a^ | 95% CI^b^ |
| Age |  |  |  |  |
| 40-49 | 1 | - | 1 | - |
| 50-59 | 1.56* | [1.29-1.88] | 1.10 | [0.78-1.54] |
| 60-69 | 3.09* | [2.50-3.83] | 0.74 | [0.53-1.04] |
| 70+ | 6.03* | [4.63-7.87] | 0.70 | [0.48-1.02] |
| Gender^c^ | 1.44* | [1.22-1.69] | 1.16 | [0.88-1.53] |
| Wealth quintile |  |  |  |  |
| 1^d^ | 1 | - | 1 | - |
| 2 | 0.96 | [0.75-1.21] | 1.00 | [0.69-1.47] |
| 3 | 1.11 | [0.88-1.41] | 1.64* | [1.11-2.44] |
| 4 | 1.16 | [0.91-1.46] | 1.07 | [0.74-1.55] |
| 5^e^ | 1.25 | [0.99-1.59] | 0.98 | [0.68-1.41] |
| Any education^f^ | 1.01 | [0.81-1.26] | 1.05 | [0.72-1.52] |
| Married / cohabiting^g^ | 0.76* | [0.63-0.92] | 1.49* | [1.12-2.00] |
| *p<0.05  ^a^ Odds Ratio. ^b^ confidence interval. ^c^ reference: male. ^d^ poorest. ^e^ wealthiest. ^f^ reference: no formal education. ^g^ reference: not being married or cohabiting | | | | |

R**EFERENCES**

Brinkmann, B., Payne, C. F., Kohler, I., Harling, G., Davies, J., Witham, M., . . . Bärnighausen, T. (2020). Depressive symptoms and cardiovascular disease: a population-based study of older adults in rural Burkina Faso. *BMJ open, 10*(12), e038199. doi:10.1136/bmjopen-2020-038199

Filmer, D., & Pritchett, L. H. (2001). Estimating Wealth Effects Without Expenditure Data—Or Tears: An Application To Educational Enrollments In States Of India*. *Demography, 38*(1), 115-132. doi:10.1353/dem.2001.0003

Guerchet, M., Mbelesso, P., Ndamba-Bandzouzi, B., Pilleron, S., Desormais, I., Lacroix, P., . . . Preux, P.-M. (2014). Epidemiology of dementia in Central Africa (EPIDEMCA): protocol for a multicentre population-based study in rural and urban areas of the Central African Republic and the Republic of Congo. *SpringerPlus, 3*(1), 1044. doi:10.1186/2193-1801-3-338

Hall, K., Hendrie, H., Brittain, H., Norton, J., Rodgers, D., Prince, C., . . . Nath, A. (1993). The development of a dementia screeing interview in two distinct languages.

Kolenikov, S., & Angeles, G. (2009). Socioeconomic Status Measurement With Discrete Proxy Variables: Is Principal Component Analysis A Reliable Answer? *Review of Income and Wealth, 55*(1), 128-165. doi:10.1111/j.1475-4991.2008.00309.x

Kroenke, K., Spitzer, R. L., Williams, J. B., & Lowe, B. (2010). The Patient Health Questionnaire Somatic, Anxiety, and Depressive Symptom Scales: a systematic review. *Gen Hosp Psychiatry, 32*(4), 345-359. doi:10.1016/j.genhosppsych.2010.03.006

Prince, M., Acosta, D., Ferri, C. P., Guerra, M., Huang, Y., Jacob, K. S., . . . Dementia, G. (2011). A brief dementia screener suitable for use by non-specialists in resource poor settings--the cross-cultural derivation and validation of the brief Community Screening Instrument for Dementia. *International journal of geriatric psychiatry, 26*(9), 899-907. doi:10.1002/gps.2622

Rutstein, S. O., & Johnson, K. (2004). *The DHS wealth index*. Retrieved from Calverton, Maryland, USA: <http://dhsprogram.com/pubs/pdf/CR6/CR6.pdf>
